# Anaphylaxis-related Malpractice Lawsuits

**DOI:** 10.5811/westjem.2018.4.37453

**Published:** 2018-06-04

**Authors:** Rachel A. Lindor, Erika M. McMahon, Joseph P. Wood, Annie T. Sadosty, Eric T. Boie, Ronna L. Campbell

**Affiliations:** * Mayo Clinic, Department of Emergency Medicine, Rochester, Minnesota; † Mayo Clinic, Department of Emergency Medicine, Phoenix, Arizona

## Abstract

**Introduction:**

Anaphylaxis continues to cause significant morbidity and mortality. Healthcare providers struggle to promptly recognize and appropriately treat anaphylaxis patients. The goal of this study was to characterize anaphylaxis-related malpractice lawsuits.

**Methods:**

We collected jury verdicts, settlements, and court opinions regarding alleged medical malpractice involving anaphylaxis from May 2011 through May 2016 from an online legal database (Thomson Reuters Westlaw). Data were abstracted onto a standardized data form.

**Results:**

We identified 30 anaphylaxis-related malpractice lawsuits. In 80% of cases, the trigger was iatrogenic (40% intravenous [IV] contrast, 33% medications, 7% latex). Sixteen (53%) cases resulted in death, 7 (23%) in permanent cardiac and/or neurologic damage, and 7 (23%) in less severe outcomes. Fourteen (47%) of the lawsuits were related to exposure to a known trigger. Delayed recognition or treatment was cited in 12 (40%) cases and inappropriate IV epinephrine dosing was reported in 5 (17%) cases. Defendants were most commonly physicians (n=15, 50%) and nurses (n=5, 17%). The most common physician specialties named were radiology and primary care (n=3, 10% each), followed by emergency medicine, anesthesiology, and cardiology (n=2, 7% each). Among the 30 cases, 14 (47%) favored the defendant, 8 (37%) resulted in findings of negligence, 3 (10%) cases settled, and 5 (17%) had an unknown legal outcome.

**Conclusion:**

Additional anaphylaxis education, provision of epinephrine autoinjectors or other alternatives to reduce dosing errors, and stronger safeguards to prevent administration of known allergens would all likely reduce anaphylaxis-related patient morbidity and mortality and providers’ legal vulnerability to anaphylaxis-related lawsuits.

## INTRODUCTION

Anaphylaxis is most simply understood as a multisystem and potentially life-threatening allergic reaction.^1^ Although no universal definition for anaphylaxis exists, diagnostic criteria have been developed to help medical providers promptly recognize and initiate treatment in patients experiencing severe allergic reactions or anaphylaxis.^1^ These criteria suggest treatment is appropriate for patients who develop hypotension after exposure to a known trigger or in patients who rapidly develop symptoms involving multiple organ systems, with or without confirmed exposure to a trigger.^1^ Initial treatment of anaphylaxis requires administration of epinephrine intramuscularly (IM), with use of intravenous (IV) epinephrine reserved for cases that are refractory to IM epinephrine and IV fluids. Other medications, such as antihistamines and steroids, are recommended as adjunctive, second-line therapies. Timely treatment is exceedingly important as the median time between exposure to cardiopulmonary arrest in fatal cases ranges from just five minutes in cases of medication reactions, to 15 minutes for insect stings, and 30 minutes for food.^2^

Although the dangers of anaphylaxis have been well recognized for over a century, patients with anaphylaxis are consistently underdiagnosed and inappropriately treated.^3^ Providers frequently fail to both recognize anaphylaxis and to treat patients with the correct dose of epinephrine, often struggling with the different formulations of epinephrine.^4^, ^5^ This has been best studied among radiologists and emergency physicians, who manage the majority of acute cases, but is almost certainly true for a broader range of medical providers.^3^, ^6^, ^7^ These delayed diagnoses and inappropriate treatments contribute to the estimated 1,500 deaths caused by anaphylaxis each year in the United States alone.^7^

This study seeks to characterize the incidence, patient characteristics, and legal outcomes of cases in which healthcare providers were sued for their alleged mismanagement of patients with anaphylaxis. Our goal was to highlight these legal risks to serve as additional evidence for providers that knowledge of anaphylaxis diagnosis and management is essential in a broad range of clinical specialties and settings.

## METHODS

### Study Design

We searched an online subscription legal database (Thomson Reuters Westlaw) for all relevant court opinions, jury verdicts, and settlements, using a Boolean search of malpractice cases with the query terms starting with “anaphyla-,” or “allergi-.” We excluded cases with the term “Eighth amendment” as there were a significant number of cases not relevant to this study involving prisoners’ claims that their Eighth Amendment rights had been violated due to failure to provide accommodations for their allergies. We included in this study all cases reported in the five-year period from May 15, 2011, through May 15, 2016. No medical records were accessed. This search strategy was similar to that used in previous legal case series and was exempted from review by the institutional review board.^8^, ^9^

### Data Collection and Primary Data Analysis

Of the 327 unique cases identified in the initial search, most cases were excluded because they were unrelated to an allergic reaction or anaphylaxis. The most common reasons for exclusion were cases involving adverse rather than allergic reactions (n=32), allergic reactions that occurred outside of the medical context such as in restaurants or schools (n=22), or mild allergic reactions that did not qualify as anaphylaxis (n=15). Overall, the search yielded 30 unique cases alleging medical malpractice against providers regarding cases of anaphylaxis.

Per recognized chart-review methods,^10^ we created a standardized data collection form to record patient and provider demographics, nature of the trigger, clinical management, and medical and legal outcomes. Two primary abstractors (RAL, EMM) piloted the data collection form by independently abstracting five full cases. Ambiguities in data collection were clarified with the entire investigative team. The two abstractors then independently abstracted the information for all 30 cases, with a senior investigator (RLC) adjudicating any disagreements or ambiguous data. Categorical data are presented as frequency of occurrence, and continuous data are summarized as means and ranges.

**Population Health Research Capsule**

What do we already know about this issue?

Patients with anaphylaxis are frequently underdiagnosed and inappropriately treated in many healthcare settings.

What was the research question?

What are the causes and outcomes of anaphylaxis related medical malpractice lawsuits?

What was the major finding of the study?

Delayed recognition, inappropriate treatment, and known allergen exposures are major causes of anaphylaxis related lawsuits.

How does this improve population health?

Additional provider education, use of epinephrine autoinjectors, and safeguards to prevent known trigger exposure would decrease anaphylaxis-related patient morbidity and mortality.

## RESULTS

Table 1 summarizes the 30 cases involving malpractice lawsuits related to anaphylaxis. Additional details are found in Table 2.

### Patient Characteristics and Outcomes

The majority of patients were females (n=22; 73%). Three (10%) of the cases involved pediatric patients. The most common inciting trigger was IV contrast, which was involved in 12 (40%) of the cases. Medications were the second most common trigger, resulting in anaphylaxis in 10 (33%) of the cases. The vast majority of the cases involved severe reactions with poor outcomes. Sixteen (53%) of the cases resulted in death, five (17%) in permanent neurologic damage, four (13%) in an intensive care unit (ICU) admission, and four (13%) in non-fatal cardiac arrest. Seven of the 16 deaths (44%) were related to exposure to a trigger to which the patient had a known allergy (three IV contrast, two food, one medication, one latex). The remaining nine deaths (56%) were attributed to delayed or inadequate treatment or inadequate pre-treatment for IV contrast. There were no deaths attributed to inappropriate administration of IV epinephrine; however, two of the five patients who received inappropriate doses of IV epinephrine had permanent cardiac dysfunction, one patient had both permanent cardiac and neurologic dysfunction, and two patients required ICU admission without reported long-term morbidity.

### Legal case characteristics and outcomes

Nearly half of the lawsuits (n=14; 47%) were related to exposure to a known trigger. Delayed recognition and treatment was cited in 12 (40%) cases, and inappropriate epinephrine dosing was reported in five (17%) cases. All of the cases of inappropriate epinephrine dosing were due to IV rather than IM administration of epinephrine. In one case the patient received 10 times the recommended dose of epinephrine as a result of confusion over route and concentration.

Among the 30 cases, 14 (47%) were decided in favor of the defendant, 8 (27%) resulted in findings of negligence, 3 (10%) cases settled, and 5 (n=17%) had an unknown legal outcome. The mean award amount in cases ending in findings of negligence was $1.4 million, compared to just over $375,000 for cases that settled. The most commonly named defendants were physicians (n=15, 50%) and nurses (n=5, 17%). The most common physician specialties named were radiology and primary care (n=3, 10% each), followed by emergency medicine, anesthesiology, and cardiology (n=2, 7% each).

## DISCUSSION

In this review of five years of case law, we identified 30 lawsuits against healthcare providers related to anaphylaxis. The most common cause of the lawsuits was exposure to a known trigger followed by delayed recognition or treatment of anaphylaxis and inappropriate use of IV epinephrine, including both over- and under-dosing errors. Seventy-seven percent of the cases resulted in death or permanent neurologic or cardiac dysfunction. The healthcare providers involved in the lawsuits were from multiple specialties and healthcare settings, demonstrating the need for all providers to know how to recognize and treat anaphylaxis.

Many cases in this series (40%) revolved around providers’ failure to recognize and treat anaphylaxis in a timely manner. The difficulty in diagnosing anaphylaxis in the acute setting has been well recognized for many years, exacerbated by previous definitions that focused largely on underlying mechanisms and physiological responses rather than clinical signs and symptoms.^11^ The difficulty in applying these definitions to patients in acute care settings led to the development of clinical criteria to help providers identify patients with anaphylaxis within the first few minutes of assessment.^1^ Despite the fact that these clinical criteria were endorsed over a decade ago and accompanied by clear instructions for management, evidence continues to demonstrate that anaphylaxis remains under-recognized and under-treated.^12^ Our results suggest that this may be the case in a broad range of healthcare settings and highlights the need for all healthcare providers to be able to recognize and treat anaphylaxis expeditiously.

Beyond recognition of anaphylaxis, the appropriate administration of epinephrine has proven to be an additional and pervasive challenge for providers. Providers’ discomfort with epinephrine dosing has been demonstrated in multiple countries and specialties including radiology, internal medicine, emergency medicine, and pediatrics.^6^, ^13^, ^14^, ^15^ In the emergency department (ED) setting, for example, among patients with severe allergic reactions or anaphylaxis—all of whom should receive epinephrine as first-line treatment—less than one quarter actually received any epinephrine in any form.^16^, ^3^ In a survey of over 250 North American radiologists, no radiologist was able to correctly identify the preferred dose and route of administration of epinephrine for patients with anaphylaxis, and only 11% knew which concentration of epinephrine was available to them in their own institution.^6^ These numbers suggest a need to prioritize epinephrine-related education for providers, especially for those who routinely oversee the administration of medications and IV contrast.

Equally problematic to inadequate epinephrine dosing is the use of overly aggressive IV epinephrine dosing. In a literature review of complications of epinephrine administration in an ED setting, all identified cases involved IV rather than IM epinephrine, with most of these resulting in cardiac injury.^17^ In our study, 17% of the lawsuits were related to inappropriate administration of IV epinephrine complicated by non-fatal cardiac arrest as well as permanent cardiac and neurologic dysfunction. The use of IV bolus epinephrine in patients presenting to an ED has been shown to be associated with a 61 times higher risk of overdose when compared to IM administration; furthermore, three-fourths of the IV epinephrine overdoses were associated with adverse cardiovascular events including cardiac ischemia and ventricular tachycardia.^5^ Notably, the majority of these epinephrine overdoses occurred prior to ED arrival, including in post-operative areas, infusion therapy centers, and by prehospital emergency medical responders.^5^ Radiologists have also demonstrated difficulty with epinephrine dosing; those surveyed about appropriate management of contrast-induced anaphylaxis selected epinephrine dosing that would have been a significant overdose in 17% of cases,^6^ and in another study 42% of patients actually treated with IV epinephrine for a contrast reaction received an overdose.^18^

The availability of epinephrine autoinjectors may be one option to mitigate provider reluctance to administer epinephrine and decrease dosing errors. The introduction of epinephrine autoinjectors along with an anaphylaxis management order set was shown to increase the use of epinephrine in a study of ED anaphylaxis management.^19^ In addition, a recent survey study of ED healthcare providers demonstrated that autoinjector administration of epinephrine was preferred to manual epinephrine injection and believed to reduce the risk of dosing errors.^20^ The use of prefilled epinephrine syringes has also been suggested as an alternative to the more costly commercially manufactured autoinjectors, and the stability and sterility of the epinephrine has been demonstrated at three months after the preparation.^21^

Inadvertent exposure to a known allergen was the leading cause of lawsuits and the leading cause of patient death in this study. Exposures to triggers to which a patient has a known allergy represent avoidable medical errors, and healthcare institutions must continue to implement systems to avoid these errors. Specific systems designed to address these avoidable errors are beyond the scope of this paper. Medications, including IV contrast, have been demonstrated to be a leading cause of fatal anaphylaxis, as they were in this study.^2^, ^22^, ^23^ This is likely due to a more rapid onset of cardiopulmonary arrest with medication exposure, with a median time of five minutes in cases of fatal anaphylaxis, compared to 15 and 30 minutes for food and insect stings, respectively.^2^ This underscores the need for healthcare facilities, particularly radiology departments, to have protocols in place to rapidly and safely treat iatrogenic anaphylaxis.

## LIMITATIONS

This study is limited based on its reliance on court opinions as the primary source of data. No medical records were accessed. Court opinions are written by judges, court reporters, or other employees of the court with no standardized reporting formats, and therefore they include widely varying amounts of detail. As a result, certain pieces of information that may be relevant to clinicians were often not available in these reports and are missing from our data. In addition, although the legal database used contains tens of thousands of cases, it is not a comprehensive database of all legal cases; no such data source exists. Instead, the database is a combination of cases that have been appealed and a selection of trial court cases and settlements chosen for inclusion by individual court reporters. Consequently, the cases here provide descriptive data for a subset of anaphylaxis-related cases, not a comprehensive list of all lawsuits that occurred during our study period.

## CONCLUSION

Our data suggest several possible lessons for moving forward. First, despite significant progress in the development of clinical criteria to facilitate prompt recognition and treatment of patients with anaphylaxis, providers continue to struggle in this realm, suggesting the need for additional education on this topic. The diversity of provider types and range of affected specialties are compelling, emphasizing the need for this education to be directed at a similarly broad range of providers, specifically to help them quickly identify when epinephrine is needed. Second, the inappropriate use and consequent morbidity and mortality associated with IV epinephrine in this study reflect the dangers of IV epinephrine demonstrated in previous studies; this leads us to echo prior recommendations to make epinephrine autoinjectors or other lower cost alternatives available, rather than relying on providers to navigate the different epinephrine formulations found in many acute care settings.

Finally, exposure to known triggers was a common problem in our cases and highlights the need for continued systems improvements to reduce these avoidable errors. These three interventions—additional provider education in a broad range of healthcare settings regarding recognition and management of anaphylaxis; provision of epinephrine autoinjectors or other alternatives to reduce doing errors; and stronger safeguards to prevent exposure to known triggers—would all likely decrease the patient morbidity and mortality associated with anaphylaxis as well as reduce providers’ legal vulnerability to anaphylaxis-related lawsuits.

## Figures and Tables

**Table 1:** Characteristics of cases, patients, and outcomes of 30 lawsuits related to anaphylaxis.

	N (%)
Patient demographics	
Female	22 (73%)
Male	8 (27%)
Pediatric patient (age <18 yrs)	3 (10%)
Inciting trigger	
IV contrast	12 (40%)
Latex	2 (7%)
Cephalosporin	2 (7%)
Other medication	8 (27%)
Food	2 (7%)
Insect sting	2 (7%)
Not reported	2 (7%)
Patient outcome^a^	
Death	16 (53%)
Permanent neurologic damage^b^	5 (17%)
Permanent cardiac dysfunction	4 (13%)
Non-fatal cardiac arrest	4 (13%)
ICU admission	4 (13%)
Other severe reaction (hospitalization, long-term consequences)	3 (10%)
Defendant named in lawsuit^c^	
Physician	15 (50%)
Hospital	13(43%)
Nurse	5 (17%)
Other (clinic, radiology technician, school, EMS, rehab facility)	6 (20%)
Physician specialty (if specified)^c^	
Radiology	3 (10%)
Primary care (internal medicine, family medicine)	3 (10%)
Emergency medicine	2 (7%)
Anesthesiology	2 (7%)
Cardiology	2 (7%)
Other (plastic surgery, otolaryngology, urology, ophthalmology, neurology, obstetrics)	6 (20%)
Reason for lawsuit^d^	
Exposure to known trigger	14 (47%)
Delayed diagnosis/inadequate treatment	12 (40%)
Inappropriate administration of IV epinephrine	5 (17%)
Inadequate pretreatment for contrast	3 (10%)
Outcome of lawsuit	
No liability	14 (47%)
Negligence	8 (27%)
Settlement	3 (10%)
Unknown	5 (17%)
Amount of settlement/judgment	Mean (range)
Cases ending in finding of negligence	$1,407,368 ($27,500 – 4,500,000)
Cases ending in settlement	$376,667 ($250,000 – 440,000)

*IV,* intravenous; *ICU*, intensive care unit; *EMS*, emergency medical services.

a Some patients had more than one outcome.

b Includes case in which permanent neurologic injury was caused to baby in utero allegedly from maternal hypotension leading to fetal hypoxia.

c Some cases named more than one defendant or specialty.

d Some cases had more than one reason for the lawsuit.

**Table 2:** Summary table of Individual legal cases related to anaphylaxis.

Year of report	Legal outcome ($ amount)	Trigger	Defendant	Patient outcome	Reason(s) for lawsuit
2016	No Liability	Medication (cephalosporin)	Hospital	Anaphylaxis and hospitalization	aExposure (prior cephalosporin allergic reaction)
2015	No Liability	Medication (ranitidine)	Hospital	bHypoxic brain injury of fetus resulting in permanent neurologic dysfunction	Exposure (known allergy)
2015	No Liability	IV Contrast	Hospital and radiology technician	Death	Failure to identify risk factors for allergic reaction
2015	Negligence ($3,615,000)	IV Contrast	ED physicians, OB physician, and hospital	Permanent neurologic dysfunction	Exposure (known allergy)
2015	Negligence ($842,340)	IV Contrast	Radiologist	Fall, disfigurement, and disability	Inadequate treatment
2014	No Liability	IV Contrast	Radiologist	Cardiac arrest, permanent cardiac and neurologic dysfunction	Inadequate treatment (delayed)
2014	No Liability	Medication (cephalosporin)	Clinic and provider	Death	Inadequate treatment (delayed)
2014	Negligence ($4,500,000)	IV Contrast (MRI)	Neurologist	Death	Exposure (known allergy) and inadequate treatment
2014	Unknown	Bee sting	Hospital and emergency department nurse	Permanent cardiac dysfunction	Inappropriate IV epinephrine
2014	No Liability	Not reported	School district and nurse	Death	Inadequate treatment (no epinephrine)
2014	Unknown	Latex	Hospital, otolaryngologist, anesthesiologist	ICU admission	Exposure (known allergy)
2014	No Liability	Medication (acetaminophen)	Hospital	ICU admission	Inappropriate IV epinephrine
2014	No Liability	Medication (morphine)	Emergency medical services company	Death	Inadequate treatment (epinephrine after cardiac arrest)
2013	Unknown	IV Contrast	Hospital, physician	Death	Exposure (known allergy)
2013	No Liability	Food (blueberries)	School nurse, school, city	Death	Exposure (known allergy), inadequate treatment (epinephrine delayed)
2013	Unknown	Medication (methylprednisolone)	Home infusion nurse	Death	Inadequate treatment (no epinephrine available)
2013	Negligence ($375,000)	IV Contrast	Family med physician/clinic	Death	Inadequate treatment (delayed)
2013	No Liability	IV Contrast	Ophthalmologist	Death	Failure to premedicate patient with “iodine allergy,” inadequate treatment
2013	Negligence ($430,000)	Medication (Vicodin)	Urologist and hospital	Death	Exposure (to oxycodone) and inadequate treatment (delayed)
2013	Settlement ($440,000)	Not reported	Not reported	Cardiac arrest, permanent neurologic and cardiac dysfunction	Inappropriate IV epinephrine
2012	Settlement ($250,000)	Food (chocolate)	Rehabilitation facility	Death	Exposure (known allergy)
2012	Negligence ($1,000,000)	IV Contrast	Cardiologist	Death	Exposure (inadequate pretreatment for known contrast allergy)
2012	Settlement ($440,000)	IV Contrast	Radiologist	Cardiac arrest, permanent cardiomyopathy	Inappropriate IV epinephrine
2012	Unknown	IV Contrast	Internist, cardiologist, hospital	Death	Failure to premedicate patient with shellfish allergy
2012	No Liability	IV Contrast	Hospital	Debilitating fatigue	Exposure (known allergy)
2012	Negligence ($27,500)	Bee sting	Hospital	ICU admission	Inappropriate IV epinephrine
2011	Negligence ($4,691,000)	Latex	Hospital and surgical nurses	Death	Exposure (known allergy)
2011	No Liability	Medication (NSAID)	Emergency physician	ICU admission	Exposure (known allergy)
2011	No Liability	Medication (lidocaine)	Plastic surgeon	Cardiac arrest, permanent cardiac and neurologic damage	Exposure (known allergy)
2011	No Liability	Medication (not specified)	Anesthesiologist	Death	Delayed airway intervention

*IV*, intravenous; *ICU*, intensive care unit; *MRI*, magnetic resonance imaging; *ED*, emergency department; *OB*, obstetrics; *NSAID*, nonsteroidal anti-inflammatory drug.

a Patient’s prior medication allergy had been inappropriately documented.

b Secondary to maternal hypotension.

Exposure indicates exposure to substance to which the patient had had a prior allergic reaction.
